# 

**Published:** 1866-05

**Authors:** 


### CONTENTS.

### ORIGINAL CONTRIBUTIONS.

ART. XVI. A New Theory of the Origin of "Dumb-Bell" Crystals. By D. W. Flora, M.D., Chicago, ---- 257

ART. XVII. Practical Remarks on Blood-Letting. By D. B. Trimble, M.D., Chicago, ---- 262

ART. XVIII. Death from Tubal Pregnancy. By A. Fisher, M.D., Chicago, ---- 270

### SELECTIONS.

Lectures on the Recent Advances of our Knowledge in the Diagnosis and Treatment of Functional Nervous Affections. By C. E. Brown-Sequard, M.D., F.R.S., &c., ---- 272

On the Influence of the Ingestion of Coffee on the Urea and Chlorides in the Urine. By Chas. E. Squarey, M.R.C.S., Resident Medical Officer to the London Fever Hospital. Communicated by Dr. Garrod, ---- 288

Lectures on Cholera. By Prof. Alonzo Clark, M.D., ---- 292

Medicinal Uses of Ptelea Trifoliata. By O. F. Potter, M.D., St. Louis, ---- 298

Asiatic Cholera in Virginia, ---- 299

### PROCEEDINGS OF SOCIETIES.

Report to the Chicago Academy of Sciences.—Trichinæ, ---- 300

DeWitt County Medical Society, ---- 310

### EDITORIAL.

Convention of Representatives from Medical Colleges of the West, ---- 312

Cholera, ---- 317

Trichina, ---- 315

Illinois State Medical Society, ---- 317

Notice to Subscribers, ---- 317

Books Received:—

A Manual of the Principles of Surgery, based on Pathology. For Students. By Wm. Canniff, Licentiate of the Medical Board of U. C., ---- 317

Diarrhœa and Cholera; their Origin, Cause, and Cure by Means of Ice. By John Chapman, M.D., etc., etc., ---- 317

